# Marine Demospongiae: A Challenging Treasure of Bioactive Compounds

**DOI:** 10.3390/md20040244

**Published:** 2022-03-31

**Authors:** Roberta Esposito, Serena Federico, Marco Bertolino, Valerio Zupo, Maria Costantini

**Affiliations:** 1 Department of Ecosustainable Marine Biotechnology, Stazione Zoologica Anton Dohrn, Villa Comunale, 80121 Napoli, Italy; roberta.esposito@szn.it (R.E.); serena.federico@szn.it (S.F.); 2 Department of Biology, University of Naples Federico II, Complesso Universitario di Monte Sant’Angelo, Via Cin-thia 21, 80126 Naples, Italy; 3 Department of Earth, Environment and Life Sciences (DISTAV), Università degli Studi di Genova, Corso Europa 26, 16132 Genova, Italy; marco.bertolino@unige.it

**Keywords:** Demospongiae, bacteria, fungi, diverse bioactivities

## Abstract

In the last decades, it has been demonstrated that marine organisms are a substantial source of bioactive compounds with possible biotechnological applications. Marine sponges, in particular those belonging to the class of Demospongiae, have been considered among the most interesting invertebrates for their biotechnological potential. In this review, particular attention is devoted to natural compounds/extracts isolated from Demospongiae and their associated microorganisms with important biological activities for pharmacological applications such as antiviral, anticancer, antifouling, antimicrobial, antiplasmodial, antifungal and antioxidant. The data here presented show that this class of sponges is an exciting source of compounds, which are worth developing into new drugs, such as avarol, a hydroquinone isolated from the marine sponge *Disidea avara*, which is used as an antitumor, antimicrobial and antiviral drug.

## 1. Introduction

### 1.1. Natural Products from Marine Organisms

The discovery of marine-derived natural products is a promising, comparatively new field, which started with the isolation of unusual nucleoside derivatives in the sponge *Tectitethya crypta* (de Laubenfels, 1949) (ex. *Tethya crypta*) in the 1950s by Bergmann and Feeney [1,2]. In the early 1960s, research on marine natural products was driven by chemical studies and a few compounds were tested for relevant bioactivity [3]. An example is represented by the production of a pyrrole antibiotic by a marine bacterium *Pseudomonas bromoutilis* [4]. However, the utilization of marine organisms as sources of bioactive metabolites started seriously at the end of 1960s [5] with the isolation of prostaglandin derivatives from the Caribbean gorgonian *Plexaura homomalla* (Esper, 1794) [6]. In the 80s, effective collaborations were established between marine chemists and pharmacologists, and the investigations were focused on the toxins active in the membranes of the central nervous system, ion channel effectors, anticancer and antiviral agents, iser promoters and anti-inflammatory agents [7]. In the 90s, the pharmaceutical and biotechnological industries focused on the chemical libraries of both natural products and the synthetic compounds produced by combinatorial methods [8]. Invertebrates, mainly sponges, tunicates, bryozoans and shellfish, provided several marine natural products, which could be used for clinical or preclinical studies [9]. In fact, the research led to the discovery of many anticancer compounds from marine sponges, which have not yet been tested on humans, except for the Eribulin mesylate (an analogue of halichondrin B), which has been tested on women with breast cancer [10]. Many clinical trials were made through experimental models, such as the mice and zebrafish, representing a step forward in the evaluation of possible adverse effects not detectable by in in vitro tests [11]. The discovery of marine natural products has accelerated in the last two decades with the number of new compounds discovered each year increasing from 20 to more than 200 [12]. It has been assessed that more than 15,000 marine natural products (MNPs) have been discovered [13,14,15] since 2010, with 8368 new compounds recorded in the decade of 2001–2010. This constitutes over half of all the compounds discovered since 1951 [16]. Among the marine organisms investigated, marine sponges (Porifera) are recognized as the richest sources of MNPs, with about 9398 compounds to date, contributing to nearly 30.7% of all marine natural products discovered so far, according to a database of MNPs [17,18,19,20,21,22]. This makes sponges the most prolific marine producers of compounds, with more than 200 new compounds reported each year in the last decade [23]. With this myriad of MNPs already available, several studies have revealed a broad spectrum of biological activities for these compounds, including anticancer, antiviral, antibacterial, antifungal, antiprotozoal, antihelmintic, anti-inflammatory, immunosuppressive, neurosuppressive, neuroprotective, antifouling and a range of other bioactivities [14]. In addition, as infectious microorganisms evolve and develop resistance to existing pharmaceuticals, marine sponges are providing novel leads against bacterial, fungal and viral diseases [23,24]. The annual discovery of marine natural products continued at a constant level of about 500 products in the late 1990s [12], but this number increased from 600 to over 1000 compounds per year from 2008 to 2010, a significant increase which was partly driven by new developments in modern analytical technology and instruments, especially the development of the high resolution nuclear magnetic resonance (NMR) and mass spectrometry (MS) coupled with high-performance liquid chromatography (LC) and gas chromatography (GC) [12]. 

Several efforts have been made in the last years to identify antitumor compounds for therapeutic applications, for example, screening methods at the National Cancer Institute (NCI-NIH/USA), which aimed at identifying antitumor agents with selective cytotoxic activity against tumor cells [25]. Furthermore, several companies, as in the case of PharmaMar, performed the pharmacological evaluation and, ultimately, the commercialization of bioactive compounds [26]. 

In this review, we analyzed a set of scientific publications on sponges and sponge symbiont-related compounds that demonstrate interesting biotechnological applications in pharmacological field. In particular, we focused on Demospongiae, which are the largest class, encompassing 81% of all living sponges with almost 7,000 species worldwide [27]. Considering the abundance of molecules isolated from Demospongiae, we think that this class can be considered a challenging treasure of bioactive compounds, from which several others will be identified. 

### 1.2. Description of the Class Demospongiae

The class Demospongiae, together with Calcarea, Hexactinellida and Homoscleromorpha, belong to the phylum Porifera. Demospongiae are the class that includes the largest number of extant species [28,29]. They exhibit multiple shapes, are able to colonize any aquatic environment (marine, brackish and fresh waters) and have a wide distribution, both geographic (from polar to tropical waters) and bathymetric (from intertidal zones to depths of thousands of meters) [21]. They prefer hard substrates, but several species are also capable of living on soft bottoms, due to the presence of stems or bundles of spicules, which allow them to affix themselves to the substrate, while still remaining distant from the sediment [30,31]. Other species are able to live under the sediment, from which they release only rising, finger-like growths, ending in an osculum (habitus psammobiotic). Others, indicated as "free" specimens, are devoid of any anchoring structure and can live floating above the sediment, without attaching themselves.

Most demosponges are characterized by an aquiferous system, made of canals and choanocyte chambers (the leuconoid condition). The aquiferous system permeates the body of the sponge and pumps enough water to carry out an essential replacement. They filter heterotrophic bacteria, heterotrophic eukaryotes, phytoplankton and debris, within a size range of 0.1–50 μm. This class also includes the so called “carnivorous sponges”, which lack an aquiferous system (e.g., the family Cladorhizidae). Microorganisms that resist the sponge’s digestive process and survive its immune response can successfully inhabit the sponges [22]. Demosponges host a rich symbiotic community (Eubacteria, but also Archaea) and in some cases they reach 60% of the total biomass of the sponge [32].

Demosponge skeletons can be made up of siliceous spicules, either isolated or in conjunction with an organic collagen skeleton. Collagen can be dispersed or can give rise to sponge fibers and filaments. A few taxa, unrelated to each other, have no skeleton other than a diffused fibrillar collagen. Other minor groups have developed a hypercalcified basal skeleton with or without free spicules.

The spicules can be divided into megasclere and microsclere. The former are monaxial or tetraxial (never triaxial), while the latter is characterized by various shapes. The spicules are produced inside specialized cells (sclerocytes) and contain an organic axial filament, with a triangular or hexagonal section, around which hydrated silica are periodically deposited, giving rise to a concentric arrangement. It is generally assumed that spicule growth is a bidimensional process: the increase in length is affected by the elongation of the filament, whereas the increase in width is determined by the apposition of the silica [27]. In the class Demospongiae, the organic axial filament, which functions as a template for silica deposition, is constituted by peculiar proteins called silicateins. The potential number of spicule types in a species of sponges appears to be genetically fixed, but the environmental conditions, specifically, the availability of silicon, may determine whether a genetically determined spicule type is finally expressed [29]. In Demospongiae the cellular elements, remarkably different, are never syncytial (unlike those of the class Hexactinellida, which possess a choanosyncytium made by choanocytes fused to form a continuous cytoplasmic compartment). The reproduction of demosponges can be sexual (both oviparous and viviparous with the production of larvae, mostly of the parenchymella type) or asexual, occurring by fragmentation, budding and gemmulation.

### 1.3. Demospongiae as Sources of Beneficial Compounds

**Figure 1:**
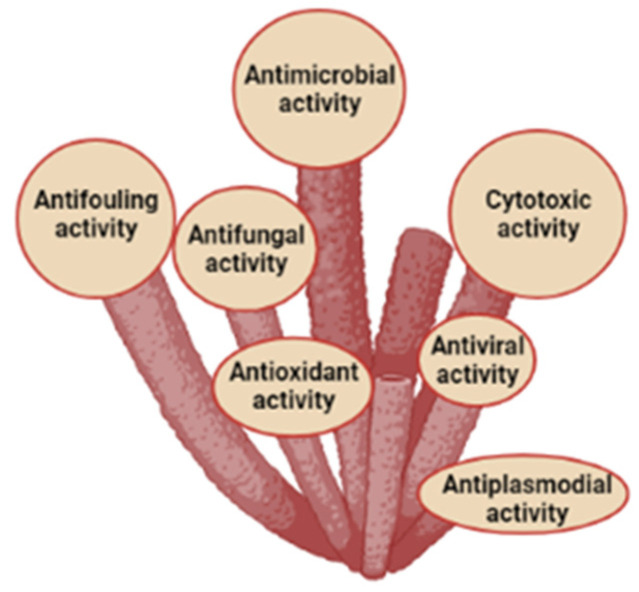
Graphical representation of sponges and their associated biota activities reported for the pharmacological application. The scale of the bubble is relative to the number of papers found. This image was created in Biorender.com (accessed on 1 January 2022).

The Demospongiae (demosponges) are the group of sponges encompassing most of the existing species, and they are an opulent source of biologically active specialized metabolites with potential biotechnological applications because of their antiviral, antitumor, antimicrobial, antiplasmodial, antifungal and antifouling [33,34,35] (see Figure 1). 

**Figure 2:**
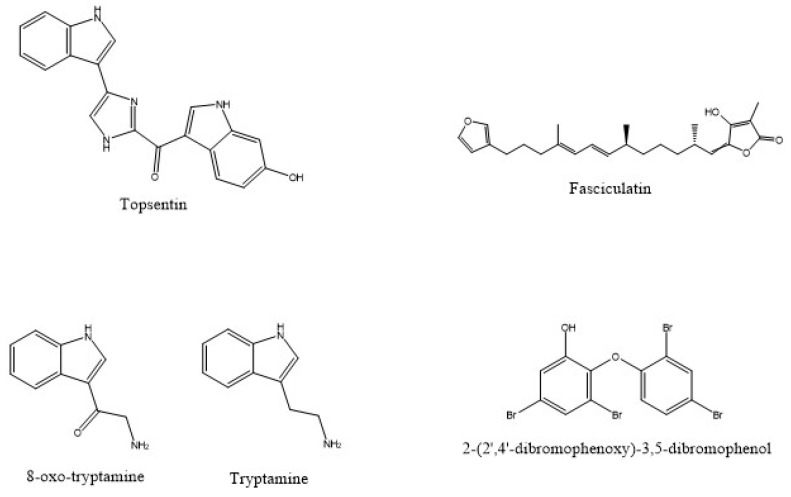
Examples of natural products isolated from some sponges belonging to the class of Demospongiae.

Specialized metabolites are not usually involved in processes like the growth, development or reproduction of an organism. They are generated as result of the organism adapting to its neighbouring environment and/or are produced to act as a possible defence mechanism against predators and to improve the fitness of the organism [36,37]. Marine natural products originate from sponges or sponges-associated biota (archaea, bacteria and fungi) [38]. Our knowledge about the heterogeneity of the sponge-associated biota is still incipient, and a large number of the features of sponge–associated biota are still unexplored. The exploration of biotechnological potentials of biota associated with sponges has been limited due to the difficulties in cultivating sponges and the microbes associated with sponges [38]. However, it is possible to perform a genome mining approach applied to all uncultured organisms to detect biosynthetic pathways of bioactive natural products, as well as their possible functional and chemical interactions [39]. In Figure 2, some examples of natural compounds isolated from the Demospongiae are depicted, which will be discussed in the following paragraphs. 

## 2. Biotechnological Activities of Compounds Isolated from Demospongiae or Their Associated Microorganisms

### 2.1. Cytotoxic Activity

Cancer is the second deadliest illness and has obtained enormous attention from researchers, who are trying to understand mechanisms of this disease and to find new drugs for therapy [40]. Marine sponges and their sponge-associated organisms represent precious sources of natural products with cytotoxic activity [41,42,43]. Two indole alkaloids, topsentin (see Figure 1) and bromotopsentin, were isolated from different species of sponges belonging to the genus *Spongosorites* (*Spongosorites* sp. and *Spongosorites ruetzleri*, Van Soest and Stentoft, 1988) and were tested on different cancer cell lines. In particular, these products showed cytotoxic activity against HCT8 (adenocarcinoma colorectal), A549 (lung carcinoma), T47D (breast carcinoma) and P388 (mouse lymphoma) with an IC_50_ of 3.0 μg/mL for the last cell line and 20 μg/mL for all other cancer cell lines [44]. Interestingly, cacospongionolide, a sesterterpene isolated from the marine sponge *Cacospongia mollior* (Schidt, 1862), collected in the northern Adriatic, showed potent antitumor activity in the brain shrimp assay with LD_50_ (lethal dose) of 0.1 μg/mL [45]. In a similar study, a polycyclic alkaloid (saraine A) isolated from the Mediterranean sponge *Reniera sarai*, previously characterized by Cimino et al. [46], tested for its cytoxic activity on the brine shrimp *Artemia salina*, showed a LD_50_ value of 46.7 μg/mL [47]. Petroleum ether and total methanolic extracts isolated from *Negombata magnifica* (Keller, 1889), collected in the Red Sea, showed anticancer activity against a human liver carcinoma cell line (HepG2) with an IC_50_ value of 5 and 10 μg/mL, respectively. Moreover, all concentrations triggered lower toxicity than positive control (palmitic acid) [48]. Similarly, aqueous ethanol extract from the marine sponge *N. magnifica*, collected along the Gulf of Aqaba in the Red Sea, had antitumor effects against MCF-7 (breast cancer) and CACO-2 (colon cancer) with an IC_50_ of 0.37 and 1.09 μg/mL, respectively [49]. Geodiamolide H3, obtained from *Geodia* sp., collected in Macqueripe Bay (Trinidad), showed in vitro cytotoxicity, calculated as total growth inhibition (TGI), against a number of human cancer cell lines: HOP-92 (non-small cell lung cancer, 0.118 μM), SF-268 (central nervous system, 0.153 μM), OV Car-4 (ovarian cancer, 0.0186 μM), A498 and UO-31 (renal cancer cells, 0.0948 μM and 0.185 μM, respectively) and MDA-MB-23 and HS 578T (breast cancer cells, 0.433 μM and 0.245 μM, respectively) [50]. Other studies on the *Geodia* genus [51] demonstrated that methanolic extracts obtained from the marine sponge *Geodia cydonium* (Jameson, 1811), collected in Gulf of Naples, manifested an anti-inflammatory effect on a MCF-7 cancer cell line, inducing a reduction in the levels of VEGF and five proinflammatory cytokines (CXCL10, CCL2, CXCL8, IFN-𝛾 and TNF-𝛼) in a dose-dependent manner. Furthermore, this extract showed a growth inhibition in three breast cancer cell lines, MDA-MB231, MCF-7 and MDA-MB468, with an IC_50_ of 44, 67 and 70 μg/mL, respectively, after 48 h of incubation [52]. Also, oxysterol and 4′-methylheptyl-benzoate isolated from the marine sponge *Hyrtios erectus* (Keller, 1889) displayed significant cytotoxic activity against breast adenocarcinoma (MCF-7) with IC_50_ values of 2.4 and 3.8 μM, respectively. The first compound, also showed an antiproliferative effect on HepG2 (hepatocellular carcinoma cells) with an IC_50_ value of 1.3 μM [53]. In an analogous study, a furanosesterterpene (fasciculation, see Figure 1) was isolated from the marine sponge *Ircinia variabilis* (Schimdt, 1862), collected from the Atlantic Coast of Morocco, and its biological activity was determined [54]. Achievements completed showed that this compound produced a dose-dependent growth inhibitory effect on MCF-7, SF-268 (CNS cancer) and NCI-H460 (non-small cell lung cancer) cell lines measured as GI_50_ (concentrations of compound, which cause 50% inhibition of tumor cell growth), corresponding to 47.11 ± 0.93, 72.45 ± 2.19 and 64.49 ± 0.84 μM, respectively, compared to the positive control doxorubicin and cyclosporin. Methanolic crude extracts from *Agelas oroides* (Schimdt, 1864) and *Petrosia ficiformis* (Poiret, 1789), collected in the Mediterranean Sea, influenced LAN5 and SK-N-BE(2)-C (human neuroblastoma cells) survival in a different way, using the concentrations of 5, 10 and 20 μg/mL of extract for 15 and 30 min. In fact, the extract of *A. oroides* was vastly more cytotoxic for two cell lines after 30 min, while the extract of *P. ficiformis* had already induced necrosis after 15 min [55]. Moreover, the cytotoxic effect of the extract from *A. oroides* differed considerably depending on seasons and depths, the greatest effect resulting from sponges collected from the site “Paraggi” in winter at −20 m [56]. In a similar work, Di Bari et al. [57] assessed the biological activity of aqueous extracts from *Tethya aurantium* (Pallas, 1766), *Tethya citrina* (Sarà & Melone, 1965), *Hymeniacidon perlevis* (Montagu, 1814), *I. variabilis*, *Chondrilla nucula* (Schimdt, 1862), *Aplysina aerophoba* (Nardo, 1843) and *Sarcotragus spinosulus* (Linnaeus, 1759), collected in the southern Adriatic Sea, on macrophages THP-1, CaCo-2 (epithelial cells), BHK-21 (fibroblasts and primary rat astrocytes) and ASTRO (astrocytes), demonstrating that the extracts from *T. citrina* and *H. perlevis* were the most cytotoxic in comparison to the other extracts analysed. In fact, ASTRO cells viability, after treatment with 30 μg/mL of extract from *T. citrina*, was of 20%, while BHK-21 cells viability treated with 30 μg/mL of extract from *H. perlevis* was 40%. Gukulenin A is a bis-tropolone tetraterpenoid obtained from the marine sponge *Phorbas gukhulensis* (Sim & Kim, 2004), which induced apoptotic cell death in A2780, SKOV3, OVCAR-3 and TOV-21G (human ovarian cancer cells) in a dose-dependent manner. The strongest cytotoxic effect was found on the ovarian carcinoma cell line A2780 at the concentration of 5 μM [58]. 

Matsumoto et al. [59] purified lectin from associated microorganisms with a black demosponge *Halichondria okadai* (Kadota, 1922), sampled in Japan. Lectins are carbohydrate-binding proteins and have many roles such as cell growth regulation, anti-infectious estates and the support of natural immunity with the help of their binding to specific oligosaccharides to create glycoconjugates. In this case, the lectin killed the Jurkat leukemia T cells and the K562 (erythroleukemia cells) in a dose-dependent manner, showing 40% and 50% cell death, respectively.

However, as mentioned in the introduction, sponge-associated biota also has a definite biotechnological role, exhibiting several bioactivities [60,61]. For instance, Pagliara and Carocco [62] isolated eight cyanobacterial strains (*Synechoccus* sp. red and blue-green types, *Cyanobium* sp., *Leptolyngbya* cfr. *minuta*, *Leptolyngbya* cfr. *ectocarpii*, *Leptolyngbya* sp. 1, 2 and 3) from *P. ficiformis*. They demonstrated that the aqueous extracts of strains, ITAC101, ITAC104 and ITAC102, belonging to *Leptolyngbya* genus, were the most toxic on *A. salina* nauplii with an LC_50_ of 6440, 10270 and 12270 μg/mL, respectively, after 24 h of exposure. Moreover, ITAC103 and ITAC104 extracts induced a delay in the development and an increment in deformed embryo production of *Paracentrotus lividus*. In the following study, Pagliara et al. [63] split eight cyanobacterial strains (*Cyanobium* sp., *Synechoccus* sp., *Pseudoanabaena* sp. 1, 2, *Leptolyngbya ectocarpi*, *Halomicronema* cf. *metazoicum*, *H. metazoicum*) isolated from the same sponge and evaluated their biological activity, testing their aqueous cell supernatants on HeLa (cervical adenocarcinoma), SH-SY5Y (neuroblastoma) and B-104-1-1 (glioblastoma). The strain ITAC106 (*Pseudanabaena* sp. 1) showed the strongest cytotoxic activity on all cell lines analysed at a concentration of 150 μg/mL. In a similar study, petrocidin A, a new cyclic dipeptide isolated from the solid culture of *Streptomyces* sp. SBT348, which had previously been recovered from the Mediterranean sponge *P. ficiformis*, displayed significant cytotoxic effects towards acute promyelocytic leukemia (HL-60) and human colon adenocarcinoma (HT-29) cell lines with the IC_50_ values of 3.9 and 5.3 μg/mL, respectively, measured with MTT assay, using as positive control 5-Flurouracil [64]. Strepoxazine A, a new phenoxazine analogue isolated from the solid culture of sponge-associated *Streptomyces* sp. SBT345, which had earlier been isolated from the Mediterranean sponge *A. oroides*, exhibited a potent cytotoxic effect against HL-60 cells (human promyelocytic leukemia) with an IC_50_ at 16 μg/mL [65]. Another study has been carried out on isolates from specimens of sponges belonging to the genus *Haliclona*. Handayani et al. [66] prepared twenty extracts of fungi derived from the marine sponge *Haliclona fascigera* (Hentschel, 1912), collected from West Sumatera, testing their biological activity using MTT (3-(4,5-dimethylthiazol-2-yl)-2,5 diphenyl tetrazolium bromide) assay and using doxorubicin as a positive control. The fungal extract of WR6 (*Trichophyton* sp.) showed the highest cytotoxicity with the IC_50_ values of 163.37, 118.3, 67.1 and 47.4, μg/mL against Vero cells, HeLa (HeLa as cervix cell line), T47D (human ductal breast epithelial tumor cell line) and WiDr (colon adenocarcinoma cell line), respectively, compared with the IC_50_ values (43.74, 1.25, 10.05 and 0.28 μg/mL, respectively) of doxorubicin. Several studies revealed the importance of kinase inhibitors from marine sponges, demonstrating the key role of these proteins in cell regulation, controlling cell differentiation, proliferation, metabolism, DNA damage repair and cell motility. The deregulation of kinase has been identified as a priority due to an ever-expanding list of diseases, including cancer, central nervous system disorders and metabolic diseases [67]. For example, penazetidine A, isolated from sponge *Penares sollasi*, and hymenialdisines 4 and 5, isolated from marine sponge *Stylotella aurantium*, exhibited activity against PKC (protein kinase C) with the IC_50_ values of 0.03, 0.8 and 1.3 μM, respectively [68,69]. The cytotoxic compounds examined above are listed in the Table 1. 

### 2.2. Antibacterial and Antiviral Activities

Specialized metabolites produced by sponges or sponge-associated biota are bioactive and indispensable for their survival in the marine environment, hence, they have potential for pharmacological applications, including antimicrobial and antiviral activities [70,71]. Sponges do not have a specific immune system but possess eosinophilic granular cells that can perform a non-specific response to a variety of dangers. This information was the foundation of the study conducted by Krylova et al. [72], which obtained pure eosinophilic amoebocyte (EA) fractions from the marine sponge *Halisarca dujardini* (Johnston, 1842), sampled from the Kandalaksha Bay, White Sea. Interestingly, only part of the subfraction showed antimicrobial activity against *Escherichia coli* and *Listeria monocytogenes* [72]. Several ethyl acetate extracts from marine sponges showed interesting activity against *E. coli*; for example, a fraction of the marine species *Aplysina fistularis* (Pallas, 1766) (sampled in Bahía de Mochima, Venezuela) with a MIC (minimum inhibitory concentration) value higher than 16 µg/mL. Instead, other fractions from the same sponge showed activity specifically on *Staphylococcus aureus* with MIC of 0.125, 128 and 256 µg/mL, respectively [73]. Similarly, manzamenones M extracted from a marine sponge belonging to the genus *Plakortis* (Okinawan Sea) showed antimicrobial activity against the same two bacterial strains (*E. coli* MIC = 32.0 µg/mL, *S. aureus* MIC = 16.0 µg/mL) and *Cryptococcus neoformans* (MIC = 4.0 µg/mL) as well. From the same sponge species another manzamenone (N) was also isolated and tested, showing biological activity against *E. coli* (MIC = 32.0 µg/mL) and *C. neoformans* (MIC = 32.0 µg/mL) [74]. Plakortide N and plakortide F free acid isolated from sponges of the genus *Plakortis* (sampled in Jamaica) showed, similarly to its Venezuelan counterpart, activity against *C. neoformans* with an IC_50_ ranging from 2.5 to 5.5 μg/mL, using amphotericin B and ciprofloxacin as positive controls [75]. 

A methanol extract from the demosponge *Xestospongia testudinaria* (Lamarck, 1815), collected in Pasir Putih (Indonesia), showed antimicrobial activity against several microbes like *S. aureus*, *E. coli*, *Klebsiella pneumoniae*, *Salmonella tiphy*, *Pseudomonas aeruginosa* MDR (Multidrug Resistant) and *S. aureus* MRSA (Methicillin resistant), using the agar diffusion method [76]. Further studies on specimens of the same sponge sampled in India demonstrated that a methanol extract of this sponge was also effective against *Staphylococcus epidermidis* [77]. Interesting compounds such as 1-monoamphilectine and 8,15- diisocyano-11(20)-amphilectene obtained from the extract of the marine sponge *Hymeniacidon* sp., sampled from the Mona Island, demonstrated their effectiveness on the *Mycobacterium tuberculosis* (H37Rv) with MIC values of 15.3 and 3.2 μg/mL [78]. 

Even though it might appear improbable, some extracts from marine sponges demonstrated higher efficiency against several Gram-positive and Gram-negative bacteria as compared to positive antibacterial positive controls. For instance, ethyl extracts from the marine sponges *Axinella damicornis* (Esper, 1794) and *A. oroides* (sampled from the Tunisian Mediterranean coast, Monastir) were more effective than the antibiotic gentamycin (10 µg) against several distinct human pathogens. *A. damicornis* demonstrated wider potentiality, as it was efficient on several bacterial strains, with a growth inhibition diameter (mm) of 21 for *S. epidermidis*, 17 for *S. aureus*, 26 *Micrococcus luteus*, and 20 for *Enterococcus feacalis*; Gram-negative bacteria: 12 for *P. aeruginosa*, 20 for *E. coli*, 16 for *Salmonella thyphymerium* and 20 for *L. monocytogenes*. In contrast, *A. oroides* was efficient only on some of those strains, with growth inhibition diameter (mm) of 13 for *S. epidermidis*, 17 for *S. aureus*, 18 for *M. luteus*, and Gram-negative bacteria 13 for *E. coli* and 12 for *S. thyphymerium*, compared with positive control gentamycin [79]. 

Powerful antibiotics were extracted and characterized from the sponge *Dysidea granulosa* (Bergquist, 1965), then tested against several different human pathogens, such as *Klebsiella pneumoniae*, with encouraging results. The recorded MIC of the compound named as 2-(2’,4’-dibromophenoxy)-3,5-dibromophenol (see Figure 1) was 0.1 μg/mL. The importance of this result is due to the fact that the positive controls available and used in the treatment of this pathogen (ciprofloxacin, cefoxitin and imipenem) are efficient at high concentrations (MIC = 0.125 μg/mL, MIC = 0.25 μg/mL and MIC= 0.25 μg/mL, respectively) [80]. Interestingly, recent studies were directed towards the extraction of compounds useful for the human health and against human pathogens from environments which are considered harsh for the humans. A pioneer study directed by Kosgahakumbura et al. [81] was focused on the extraction of bioactive compounds from the marine sponge *Stryphnus fortis* (Vosmaer, 1885). Many different peptides were purified but only one, named “peptide C”, demonstrated modest antimicrobial activity against *S. aureus* with MIC = 36.14 µM [81]. Another study was performed on two fractions, namely, A (aqueous extract) and B (methanol extract), isolated from the sponge *Suberites iona*, sampled from the Persian Arabic Gulf (PAG), which lives in hyperthermic and hypersalinic conditions. These fractions demonstrated activity not only against *S. aureus* but also against *Enterococcus faecalis* [82]. Interesting results were obtained by Tsujii and Rinehar [44], testing two indole alkaloids (topsentin and bromotopsentin) extracted from several samples of sponges belonging to the genus *Spongosorites*, collected in the Bahamas. These compounds were found to be active as antiviral agents against the *Herpes simplex* virus 1(HSV-1), vesicular stomatitis virus (VSV), and the *Coronavirus* A-59. A halistanol-enriched fraction (TSH fraction) and its compounds 1 (halistanol sulfate) and 2 (halistanol sulfate C) isolated from the sponge *Petromica citrina* (Muricy, Hajdu, Minervino, Madeira & Peixinho, 2001), collected in Brazil, showed anti-herpes activity through the reduction of viral infectivity, inhibition of virus entry into the cells and by the impairment of levels of ICP27 and gD proteins of HSV-1 [83]. In a similar study, El-Damhougy et al. [49] demonstrated that a crude extract from the marine sponge *Grayella cyathophora* (Carter, 1869), collected along the Gulf of Aqaba in the Red Sea, showed a high cytotoxic effect compared to Vero cells with the hepatitis A virus. A polycyclic alkaloids (saraine 2), isolated from sponge *R. sarai*, showed interesting antibacterial activity against *S. aureus* with MIC of 50.0 μg/mL [47].

In recent years, a number of new compounds with variegated activities have been detected through the cultivation of sponge-associated microorganisms [84,85,86]. For instance, several bacterial strains were isolated from the marine Demospongiae *Hymeniacidon perlevis*, collected on the Nanji Island (Eastern China Sea, China), and their ethyl acetate extracts were tested on human and plant pathogens, and the ones that showed significant antimicrobial activity were named as NJ6-3-1 and NJ6-3-2 and successively identified as *Pseudoalteromonas piscicida* and *Bacillus megaterium*, respectively. The extract of the former strain was efficient against *Bacillus subtilis*, *E. coli*, *S. aureus*, *Agrobacterium tumefaciens* and the yeast *Saccharomyces cerevisiae*; while the extract of the latter strain demonstrated activity against *B. subtilis*, *A. tumefaciens*, *S. aureus* and the yeast *S. cerevisiae* [87]. Frequently, the isolated bacterial species belong to the genus *Bacillus*, as is the case for the strain “2011SOCCUF3”, isolated from the marine sponge *Spongia officinales* (Linnaeus, 1759), subfractions of which were active against as *S. aureus* (MIC = 247 µg/mL), *S. tiphy* (MIC = 83 µg/mL), *P. aeruginosa* (MIC = 162 µg/mL) and *E. coli* (628 µg/mL), compared with the positive controls (ciprofloxacin and fluconazole) [88]. Similarly, from the sponge *Halichondria glabrata* (Keller, 1891) (collected in Mumbai) the strain GSA10 was isolated and successively classified for its similarities with pG1 *Bacillus amyloliquefaciens*. However, its ethyl acetate extracts were tested and were active against human pathogens such as *E. coli*, *P. aeruginosa*, *B. subtilis*, *S. aureus* [89]. In a similar way, *Bacillus* sp. Was isolated from samples of the marine sponge *Dysidea fragilis*, from the Agatti Island in the Lakshadweep archipelago, and its purified molecule (Pyrrolo(1,2-a)pyrazine-1,4-dione, hexahydro) was tested against several model bacterial pathogens, such as *Vibrio alginolyticus*, *Vibrio parahaemolyticus*, *Vibrio vulnificus*, *Flavobacterium* sp., *Proteus mirabilis* and *Citrobacter brackii*, and showed a LC_50_ of 31.25 µg/mL, using antibiotic amoxicillin as a positive control [90]. Another pathogen which affects aquaculture stocks is *Vibrio anguillarum*, isolated from the marine sponge *Erylus deficiens* (Topsent, 1927) [71]. Bacteria isolated from *P. ficiformis*, sampled from the Portofino Promontory (Ligurian Sea), were tested for their potential production of antibiotic compounds against *S. aureus*. Two strains were identified as *Rhodococcus erythropolis* and the other one belonged to the genus *Pseudomonas* [91]. Recently, Koch et al. [92] studied two sponges sampled from the North East Atlantic, *Pheronema carpenteri* (Thomson, 1869) and *Hertwigia* sp., from which several bacterial strains were isolated and tested for their antimicrobial activity. From the sponge *P. carpenter*, strains of *Bacillus altitudinis*, *Streptomyces* sp., *Brevundimonas* sp., *Microbacterium maritypicum* were isolated, while from the *Hertwigia* sp. was isolated from the species *Delftia acidovorans*. All these bacteria were active against *S. aureus*, *E. coli* and *M. luteus*. Many other bacterial strains belonging to phyla Proteobacteria, Actinobacteria, Firmicutes and Bacteroidetes were isolated from specimens of *Suberites carnosus* (Johnston, 1842) and *Leucosolenia* sp., sampled from Ireland (Lough Hyne, Co. Cork), and tested for their antimicrobial activity against many pathogens (*B. subtilis* IA40, *E. coli* NCIMB 12212, *S. aureus* NCIMB 9518, *K. marxianus* CB86556). Antibacterial activity was higher among the isolates obtained from the sponge *S. carnosus* than from the isolates separated from *Leucosolenia* sp. [93]. In a study conducted by Halloran et al. [94], three additional species of sponges (*Polymastia boletiformis* (Lamarck, 1815), *Axinella dissimilis* (Bowerbank, 1866) and *Haliclona simulans* (Johnston, 1842)) were collected from the same area. From these specimens seventy-three different bacterial strains, all belonging to *Pseudovibrio* spp., were isolated and tested for their antibacterial effect against an ample group of pathogens. The strongest antibacterial activity was discovered against *E. coli*, *S. Typhimurium*, *B. subtilis*, *S. aureus*, methicillin-resistant *Staphylococcus aureus*, *S. aureus* VISA (vancomycin intermediate), heterogenous vancomycin intermediate *Staphylococcus aureus*, *Clostridium perfringens* and *Clostridioides difficile*. A minor activity was observed when the test was carried out on *Yersinia enterocolitica*, *B. cereus*, *Enterococcus faecium*, vancomycin-resistant *Enterococcus* (VRE) and *L. monocytogenes* [94]. From samples belonging to *Ircinia* sp., collected in the North Bay (Andaman), many bacteria (*Vibrio* sp., *Bacillus* sp., *Aeromonas* sp., *Corynebacterium* sp., *Pseudomonas* sp., *Enterococcus* sp., *Streptococcus* sp., *Neisseria* sp., *Citrobacter* sp., *Veillonella* sp. and *Klebsiella* sp.) associated with this sponge were isolated and tested for their antimicrobial activity and measured by disc diffusion assay, using erythromycin and ciprofloxacin as positive controls, against Gram-negative and Gram-positive pathogens (*Aeromonas hydrophila*, *Enterococcus durans*, *Bacillus subtilis*, *Klebsiella pneumonia*, *Streptococcus lentus* and *Rolstonia solanacearum*). Most activity was recorded against Gram-positives (41.54%), whereas minor activity was found against Gram-negative bacteria (12.82%). The bacterial metabolites were differed significantly among them. Some of the isolates were specific for a single pathogen. In particular, the highest percentage was active just against 2–3 organisms, while only some isolates showed a wider spectrum of activity against 4–5 different pathogens [95]. 

Demospongiae members are not only found in the marine environment but can also be found in freshwater lakes, as is the case for the sponge *Ochridaspongia rotunda* (Arndt, 1937), an endemic species of the Lake Ohrid, in Europe. Interestingly, its methanol extract resulted in biological activity against several bacterial strains (*Enterobacter cloacae*, *E. coli*, *L. monocytogenes*, *Mariniluteicoccus flavus*, *Bacillus cereus*, *P. aeruginosa*, *Salmonella typhimurium* and *S. aureus)*, with a MIC between 7.5 and 15.0 µg/mL and MBC (minimum bactericidal concentration) between 15 and 30 µg/mL. Moreover, this extract was more effective than streptomycin and ampicillin (positive controls) [96]. The aforementioned antibacterial and antiviral activities of demosponges or demosponge-associated biota are schematically summarized in Table 2.

### 2.3. Antifouling Activity

The Woods Hole Oceanographic Institute [97] refers to fouling as the process by which “plants and animals grow on the surface of submerged artificial structures and not natural objects”. Fouling has always been the cause of worldwide economic losses by reducing boats speed and increasing fuel consumption. In addition, the losses could also be extended to aquaculture systems where fouling can erode and degrade equipment, and can also cause mass mortalities in farming plants [98,99]. Nowadays, there is the urge to implement the knowledge and study of new compounds that can replace the obsolete biocides, which were in use for a long period of time and are now banned from the market (e.g., Tributyltin and derivatives) but are still used for navy vessels [98,100]. Marine organisms such as sponges (or, indirectly, their symbionts) naturally produce antifouling compounds, which are useful to avoid larvae from marine organisms and various bacterial strains from attaching to the surface of their bodies, eventually blocking their pores and preventing filtering activity and so leading the animals to starvation [98,100]. 

Diatoms are among the organisms involved in microfouling. A study conducted by Tsoukatou et al. [101] demonstrated the antifouling activity of extracts of sponges belonging to the genus *Ircinia* on three diatom species: *Amphora coffeaformis* (AC2078), *Phaeodactylum tricornutum* (DIA12) and *Cylindrotheca closterium* (DIA6). The ability to inhibit the development of diatoms was evaluated by the addition of sponge extracts in concentrations of 30 µg/mL to a flask in which the diatoms were being cultured. Interesting results were obtained, showing that an aqueous extract of *Ircinia variabilis* was active on all three diatom species (inhibition rates varied between 1–30%), while its ethanol extract was more effective against the first two species. The ethanolic extract of another sponge belonging to the genus *Ircinia* (*I. spinosula* Schmidt, 1862) was tested on the same diatom species and demonstrated biological activity against *A. coffeaformis* (inhibition between 31–59%) and *P. tricornutum* and *C. closterium* (between 1–30%) [101]. Other monocellular organisms capable of inducing fouling are bacteria such as *Vibrio carchariae*, which may be responsible for the death of a large number of marine fish and invertebrates in aquaculture systems, leading to huge economic losses [102,103]. The MIC values of 1 µg/mL were recorded when testing tryptamine (see Figure 1) extracted from the sponge *Fascaplysinopsis reticulata* (Hentschel, 1912) on these bacteria [99]. In the same study, two novel compounds, (6-bromo-8,1’-dihydro-isoplysin A and 5,6-dibromo-8,1’-dihydro-isoplysin A), were extracted, characterized and tested against *Vibrio natrigens*, which is one of the major biofilm producers, able to corrode artificial surfaces when immersed. These two products showed encouraging activity with the MIC values of 0.01 and 1.00 µg/mL, respectively [99]. Besides *Vibrio*, other bacterial strains are involved in the fouling processes, such as *Bacillus cereus*, *Bacillus pumilus*, *Bacillus megaterium*, *Pseudoalteromonas haloplanktis*, *Pseudomonas chlororaphis*, *Pseudomonas putida* and *Pseudomonas aeruginosa*. For this reason, Mol et al. [104] investigated the effectiveness of aqueous and ethyl acetate extracts of the marine sponge *Haliclona exigua* (Kirkpatrick, 1900) on these bacterial strains (high activity at concentrations of 100 µg/disc), using penicillin-G and streptomycin as positive controls. Macroalgae are also included in the macrofouling organisms, but to date not much is known about the potentiality of using sponge extracts against macroalgae fouling. Tsoukatou et al. [101] pointed out that dichloromethane extracts from *I. oros* (Schimdt, 1864) and *I. spinosula* are the best inhibitors of the adherence of macroalgae (*Enteromorpha intestinalis*, *Ulva lactuca* and *Sargassum muticum*) to natural substrates compared with positive control TBTO (bis tributyltin oxide). 

Among the organisms that very frequently constitute macrofouling are barnacles, covering the ship hulls and their cooling system and leading to supplementary economic expenses in addition to those that are normally required for boat upkeep [98]. Marine sponges such as *Lissodendoryx isodictyalis* (Carter, 1882) (also called garlic sponge for its characteristic garlicky odour) do not present any fouling organisms on their surface. This remarkable absence has been also observed in other sponges [104,105], and this led to the idea that they are able to produce useful compounds that prevent fouling. Specimens of *L.* *isodictyalis* were collected from the Core Sound near Straits, North Carolina, at depths less than 1 meter below the low tide, and their ethyl acetate extracts were tested for settlement inhibition against the barnacle *Balanus amphitrite*. The effective concentration (EC_50_) was 100 µg/mL [105]. *L. isodictyalis* extracts were not the only ones effective against this *Balanus* species. In fact, kalihinenes X, Y and Z (diterpenes) extracted from the marine sponge Acanthella cavernosa (Dendy, 1922), collected on Yakushima Island, were also active with an EC_50_ of 0.49, 0.45 and 1.1 µg/mL, respectively [106]. In addition, from the marine sponge *Neopetrosia chaliniformis* (Thiele, 1899) (ex *Haliclona exigua*), collected in the Gulf of Mannar (India), the ethyl acetate and aqueous extracts inhibited the settlement of the B. amphitrite larvae with the EC_50_ of 6.55 µg/mL and 6.57 µg/mL, respectively [104]. Similarly, extracts of sponges belonging to *Callyspongia* spp. and Callyspongia (*Cladochalina*) plicifera (Lamarck, 1814), collected in Hong Kong and the Bahamas, respectively, showed not only activity against *B. amphitrite*, but also against the polychaete *Hydroides elegans* [107], which is another frequent fouler of boats’ hulls. In an analogous way, several sponge compounds extracted have displayed interesting activity against *Balanus improvises*, another barnacle species affecting the man-made submersed surfaces, for example barretin and 8,9-dihydrobarretin, isolated from the marine sponge *Geodia barretti* (Bowerbank, 1858), collected in the Atlantic Ocean. These natural compounds were able to inhibit the larval settlement of barnacles at concentrations of 1.9 and 19 µM, respectively [100]. Similar inhibition of barnacle larval settlement was exhibited by already known compounds such as bastadins 3, 4, and 9, extracted from the marine sponge *Ianthella basta* (Pallas, 1766), and aplysamine-2 from *Pseudoceratina purpurea* (Carter, 1880) (concentrations between 1 and 10 µM) and new compounds as Bastadin-16, hemibastadin-1 from *I. basta* and psammaplin A from *Aplysinella rhax* (de Laubenfels, 1954) inhibited larval settlement at doses of 10 µM [98]. 

Mussels are also among the most common macrofoulers which can be found attached to boats’ chains and buoys. Some examples represented by mussels, such as *Perna perna* and the acetone/dichloromethane extracts of several marine sponges collected in Brazil, including *Tethya rubra* (Samaai & Gibbons, 2005), *Tethya maza* (Selenka, 1878), *Hymeniacidon heliophila* (Wilson, 1911) and *Petromica citrina*, proved to be useful in inhibiting the power of byssus attachment with statistically significant results [108]. Furthermore, a marine sponge belonging to the genus *Haliclona*, collected in Palau, was an effective repellent against the blue mussel *Mytilus edulis and Mytilus galloprovincialis*. Specifically, this activity was attributed to two hexapeptides extracted from these sponges [109]. The compounds isolated from Demospongiae with antifouling activity examined in this section are summarized in Table 3.

### 2.4. Other Miscellaneous Activities

Due to the widespread emergence and the resistance of human pathogens to available drugs, there is a need to detect and develop new compounds. Malaria is one of the most infectious diseases in the world that frequently causes death in children and pregnant women. Rough estimates indicate 209 million cases in 2019 alone [110]. The rich and diversified marine environment has provided us with many compounds useful for biotechnological applications and for this reason it is important to once again search this enormous reservoir for antimalarial compounds and more. In fact, a few studies have been carried out on marine sponges to assess their antimalarial, antileishmanial and antitrypanosomal activities. For instance, several compounds were isolated from the marine sponge *Plakortis simplex* (Schulze, 1880) and were then tested for their antimalarial activity against two cloroquine-resistant (CQ-R) and chloroquine-sensitive (CQ-S) strains of *Plasmodium falciparum*. Besides an unknown compound, these compounds were named plakortin and plakortide Q; these three compounds exhibited consistent antimalarial activity against both strains, even though they were less efficient against the CQ-R strain [111]. In a similar study, monoamphilectine A, a diterpenoid β-lactam alkaloid, was purified from the marine sponge *Hymeniacidon* sp., sampled in Puerto Rico. It showed potent antimalarial activity with an IC_50_ value of 0.60 µM [78]. From the marine Demospongiae *Monachora unguiculata* (Dendy, 1922), collected in Madagascar, two new compounds named as ptilomycalin F and fromiamycalin were isolated, exhibiting interesting activity against *P. falciparum* with the IC_50_ values of 0.23 and 0.24 µM, respectively [112]. In a further study, a new compound named as 8-oxo-tryptamine (see Figure 1) and a mixture of two already known compounds (*E*)-6-bromo-20-demethyl-30-*N*-methylaplysinopsin and (*Z*)-6-bromo-20-demethyl-30-*N*-methylaplysinopsin, extracted from the marine sponge *F. reticulata*, exhibited antiplasmodial activity against *P. falciparum* (IC_50_ values 8.8 and 8.0 µg/mL), using artemisinin as a positive control with an IC_50_ of 0.006 ± 0.002 μg/mL. [99]. Moreover, unidentified marine bacteria were isolated from the marine sponge *Hyattella intestinalis* (Lamarck, 1814), collected in Thondi, and tested for their antiplasmodial activity. In particular, two ethyl acetate extracts of bacterial colonies named THB20 and THB34 by Inbaneson and Rayikumar [113] displayed significant antimalarial activity with the IC_50_ values of 41.88 and 42.36 µg/mL, respectively, compared with positive control chloroquine (IC_50_ of 19.59 µg/mL). A glycoprotein, named pachymatismin, isolated from the sponge *Pachymatisma johnstonii* (Bowerbank, 1842), collected along the French coast, showed cytotoxic activity with the IC_50_ of 1 µg/mL, inducing alterations in the cell shape, phospholipase A_2_ activity and the invasion capacity of the parasite (*Leishmania brazieliensis* and *L. mexicana*) [114]. This is the first compound isolated from marine sponge with antileishmanial activity. From the marine sponge *Haliclona exigua* (Kirkpatrick, 1900), from Tamil Nadu coast of India, an alkaloide named araguspongin C was isolated, demonstrating strong activity against *L. donovani* with an IC_50_ of 8.2 µg/mL in vitro and 31.2 µg/mL in vivo [115]. In another study, the compound hyrtiodoline A, isolated from the marine sponge *Hyrtios* sp., sampled from the Red Sea, exhibited potent antitrypanosomal activity against *Trypanosoma brucei* with the IC_50_ value of 7.48 µM [116].

Fungal diseases represent another increasing worldwide danger to human health. However, only a few antifungal drugs are currently available for the treatment of life-threatening fungal infections [117]. Candidosis is among the most common fungal infection in humans (accounting an estimated 40 million infections per year) affecting human mucosae. Due to its wide diffusion, this infection represents a problem in immunocompromised patients [118]. For this reason, finding novel compounds able to eliminate this pathogen and cure its infections is of fundamental importance. Sponge extracts can manifest specific activity against several strains of *Candida*. This is the case for the ethanol extracts from the sponge *A. oroides*, which was effective against *Candida albicans*, *Candida krusei*, *Candida parapsilosis*, *Candida glabrata Candida tropicalis* and *Candida dubliniensis* [79]. Similarly, Untenospongin B, extracted from the marine sponge *Hippospongia communis* (Lamarck, 1814), collected off the Atlantic coast of Morocco, exhibited interesting antifungal activity against *Candida tropicalis* (R2 CIP 1275.81), *Candida albicans* (ATCC 10231), *Fusarium oxysporum* (CIP 108.74) and *Aspergillus niger* (CIP 1082.74), using amphotericin B as a positive control [119]. Wide antifungal activity was also found in several bacterial strains isolated from two marine sponges, *S. carnosus* and *Leucosolenia* sp., sampled at a depth of 15 meters, off Lough Hyne, Co. Cork, in Ireland. All bacteria isolated were tested for their antifungal activity against *C. albicans* (Sc5314), *C. glabrata* (CBS138), *Aspergillus fumigatus* (Af293) and *Kluyveromyces marxianus* (CB86556). Most activity stemmed from the bacteria belonging to the genera *Pseudoalteromonas*, *Bacillus*, *Vibrio* and *Staphylococcus*, isolated from *Leucosolenia* sp. (15% of the total isolates), while only 4% of the bacterial strains from *S. carnosus* were active [93]. Another compound extracted from the symbiont *Bacillus sp.* (2011SOCCUF3), isolated from *Spongia officinalis* (Linnaeus, 1759), showed a strong antifungal activity. The vacuum liquid chromatography (VLC) fractions obtained from this bacterium showed specific activity against *C. albicans* (MIC = 108 µg/mL) [88]. Similarly, against *C. albicans*, a methanol extract from the marine Demospongia *Neopetrosia exigua* (Kirkpatrick, 1900) showed encouraging activity [77], as well as for the “peptide C” extracted from the deep sea marine sponge *S. fortis* (MIC = 18.07 µM [81]. A study conducted by Mohammed et al. [75] planned to test compounds of Plakortide N and Plakortide F, extracted from the sponge *Plakortis angulospigulatus* (Carter, 1879), sampled from Jamaica, on various *Candida* species (*C. glabrata*, *C. albicans*, *C. krusei*), showing IC_50_ values ranging from 0.25 to 3.0 μg/mL. Similarly, two different compounds extracted from a sponge belonging to the genus *Plakortis*, sampled from the Okinawan Sea, were named Menzamenone M and Menzamenone N. Both of them showed interesting activity against *C. albicans* [74]. Similar activity against *C. albicans* was found in 86.6% of the aqueous extracts of the bacteria belonging to Proteobacteria, Actinobacteria and Firmicutes phyla, isolated from the marine sponge *Erylus deficiens* (Topsent, 1927), using amphotericin B (0.19, 0.39, 0.78 and 1.56 μg/mL) and rifampicin (62500, 125000, 250000 and 500000 μg /mL) as positive controls [71]. 

In cases where interesting activity is not detected this does not mean that the sponge species will never develop such activity, as the production of compounds could be linked to the conditions in which the sponges are actually growing. This can be seen in a case reported by Kibungu et al. [120], where the crude extract obtained from the marine sponge *Psammaplysilla* sp., sampled from Phillips Reef, South Africa, showed seasonal variation in antifungal activity. In fact, only ethyl acetate extracts performed from sponge samples collected in spring exhibited bioactivity against *C. albicans* compared to the positive controls fluconazole, itraconazole and voriconazole. 

Sponges can also help findings in new biotechnologies for regenerative medicine. Re-generative medicine currently needs innovative biomaterials with low immunogenicity and toxicity and good mechanical properties. However, skin and bones from bovine or porcine wastes continues to be the primary source of proteins for regenerative medicine. Recently, the scientific community has shown a strong interest in the marine collagen isolated from fish and various marine invertebrates, including sponges, used in tissue engineering [121,122]. Collagen extracts from *Chondrosia reniformis* (Nardo, 1847) do not cause toxicity in mammalian cells, but positive effects on the proliferation of L929 fibroblasts, HaCat keratinocytes and RAW 264.7 macrophages. Moreover, the fractions M4 and M5 from this sponge revealed promising wound-healing properties, facilitating either cell migration or proliferation at the site of the damage to epidermal and dermal cells. So, these data suggested that extracts could be exploited for cosmetic or regenerative medicine purposes, facilitating cell migration or proliferation at the site of the wounded epidermal and dermal cells [121]. Pozzolini et al. [122] isolated collagen filaments from the marine sponges *Ircinia oros* and *Sarcotragus foetidus* (Schimdt, 1862), collected in the Ligurian Sea, and tested them on HaCat keratinocytes and L929 fibroblasts. Additionally, in this study, the extracts were effective for wound-healing when compared with the positive controls hydrogen peroxide and quartz. The products and biological activities described in this section are summarized in Table 4.

## 3. Conclusions

**Figure 3:**
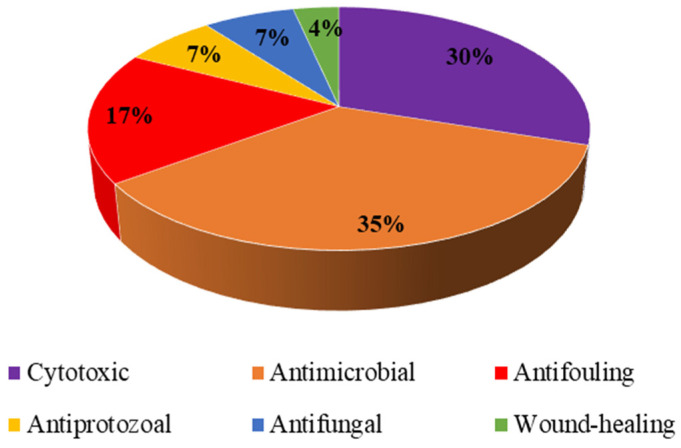
Different bioactive extracts/compounds isolated from Demospongiae.

As amply demonstrated by reviewing the available data, marine Demospongiae represent a class of sponges with great biotechnological potential. This important role in drug discovery is mainly due to the diverse range of specialized metabolites, which are isolated from different environmental and geographic conditions. In particular, the majority of natural products from Demospongiae have been isolated since 2000, so the data reported is quite recent, with a large increase in the last 10 years. Terpenes and alkaloids are the major reported chemical classes among the natural products isolated from Demospongiae, even if most reported results concern activities of total extracts or fractions not yet chemically identified. Interestingly, 35% and 30% of the compounds have properties showing antimicrobial and cytotoxic activities, respectively, as reported in Figure 3.

Another major activity seen in this class (17%), concerns the antifouling properties of some specialized metabolites, while a very low percentages of extracts/fractions demonstrate antiplasmodial, wound-healing and antifungal activities. In conclusion, the increasing number of bioactive extracts/fractions should push researchers towards additional investigation on sponges belonging to the class Demospongiae.

## Figures and Tables

**Table 1:** Source, sponge host, extract/compound, cell line/organism tested and corresponding reference are reported.

Source	Associated Organisms	Extract/Compound	Cell Line/Organism Tested	Reference
*Spongosorites* sp. and *S. ruetzleri*		Topsentin and bromotopsentin	P388, HCT8, A549, T47	[44]
*C. mollior*		Cacospongiolide	Shrimp	[45]
*R. sarai*		Saraine A	*A. salina*	[47]
*N. magnifica*		Petroleum ether and total methanolic extracts	HepG2	[48]
*N. magnifica*		Aqueous ethanol extract	CACO-2 and MCF-7	[49]
*Geodia* sp.		Geodiamolide H 3	HOP 92, SF-268, OV Car-4, A498, UO-31, MDA-MB-231, HS 578T	[50]
*G. cydonium*		Methanolic extract	MCF-7, MDA-MB231, MDA-MB468	[52]
*H. erectus*		Oxysterol and 4′-methylheptyl benzoate	MCF-7 and HepG2	[53]
*I. variabilis*		Fasciculatin	MCF-7, NCI-H460 and SF-268	[54]
*A. oroides* and *P. ficiformis*		Methanolic extract	LAN5 and SK-N-BE(2)-C	[55]
*T. aurantium*, *T. citrina*, *H.perlevis*, *I. variabilis*, *C. nucula*, *A. aerophoba and S. spinosulus*		Aqueous extract	THP-1, CaCo-2 and BHK-21	[57]
*P. gukhulensis*		Gukulenin A	A2780, SKOV3, OVCAR-3 and TOV-21G	[58]
*H. okadai*		Lectin	Jurkat leukemia T and K562	[59]
*P. ficiformis*	*Synechoccus* sp. red and blue-green types, *Cyanobium* sp., *Leptolyngbya* cfr. Minuta, *Leptolyngbya* crf. *ectocarpii*, *Leptolyngbya* sp. 1, 2 and 3	Aqueous extract	*A*. *salina* and *P. lividus*	[62]
*P. ficiformis*	*Cyanobium* sp., *Synechoccus* sp., *Pseudoanabaena* sp. 1, 2, *L. ectocarpi*, *Halomicronema* cf. *metazoicum*, *H. metazoicum*	Aqueous cell supernatans	HeLa, SH-SY5Y and B-104-1-1	[63]
*P. ficiformis*	*Streptomyces* sp. SBT348	Petrocidin A	HL-60 and HT-29	[64]
*A. oroides*	*Streptomyces* sp. SBT345	Strepoxazine A	HL-60	[65]
*H. fascigera*	*Trichophyton* sp.	Ethil acetate extract	WiDr, T47D and HeLa	[66]
*P. sollasi*		Penazetidine A	PKC	[68]
*S. aurantium*		Hymenialdisines 4 and 5	PKC	[69]

**Table 2:** Source, sponge host, extract/compound, cell line/organism tested and reference are reported.

Source	Associated Organisms	Isolated Compound	Cell Line/Organism Tested	Reference
*H. dujardini*		Eosinophilic amoebocytes (EA) fraction	*E. coli* and *L. monocytogenes*	[72]
*A. fistularis*		Ethyl acetate extract	*S. aureus*	[73]
*Plakortis* sp.		Manzamenones M and N	*E. coli*, *S. aureus* and *C. neoformans*	[74]
*P. angulospiculatus*		Plakortide N and F	*C. neoformans*	[75]
*X. testudinaria*		Methanol extract	*S. aureus*, *E.coli*, *K. Pneumoniae*, *S. tiphy*, *P. aeruginosa MDR* and *S. aureus MRSA*	[76]
*X. testudinaria*		Methanol extract	*S. epidermidis*	[77]
*Hymeniacidon* sp.		1 Monoamphilectine and 8,15-diisocyano-11(20)-amphilectene	*M. tuberculosis (H37Rv)*	[78]
*A. dormicons* and *A. orides*		Ethyl acetate extract	*S. epidermidis*, *S. aureus*, *M. luteus*, *E. feacalis*, *E. coli*, *P. Aeruginosa*, *S. thyphymerium* and *L. monocytogenes.*	[79]
*D. granulosa*		2-(2’,4’-dibromophenoxy)-3,5-dibromophenol	*K. pneumoniae*	[80]
*S. fortis*		Peptide C	*S. aureus*	[81]
*S. luna*		Aqueous extractA and methanol extract B	*S. aureus* and *E. faecalis*	[82]
*H. perleve*	*P. piscicida (NJ6-3-1) and B. megaterium (NJ6-3-2)*	Ethyl acetate extract	*B. subtilis*, *S. aureus*, *E. coli*, *A. tumefaciens* and *S. cerevisiae*	[87]
*S. officinales*	Bacillus 2011SOCCUF3	Methanol extract	*S. aureus*, *S. tiphy*, *P. aeruginosa* and *E. coli*	[88]
*H. glabrata*	*Bacillus amyloliquefaciens*	Ethyl acetate extract	*E. coli*, *P. aeruginosa*, *B. subtilis* and *S. aureus*	[89]
*D. fragilis*	*Bacillus* sp.	Pyrrolo(1,2-a)pyrazine-1,4-dione,hexahydro	*V. alginolyticus*, *V. vulnificus*, *V. parahaemolyticus*, *Flavobacterium* sp., *P. mirabilis*, *C. brackii*, *A. salmonicida* and *Edwardsiella* sp.	[90]
*E. deficiens*	Proteobacteria, Actinobacteria and Firmicutes phyla	Aqueous extract	*V. anguillarum*	[71]
*P. ficiformis*	*Rhodococcus* sp. and *Pseudomonas* sp.	Bacterial isolates	*S. aureus*	[91]
*P. carpenteri* and *Hertwigia* sp.	*B. altitudinis*, *Streptomyces* sp., *Brevundimonas* sp., *M. maritypicum* and *D. acidovorans*	Bacterial isolates	*S. aureus*, *E. coli* and *M. luteus*	[92]
*S. carnosus* and *Leucosolenia* sp.	Proteobacteria, Actinobacteria, Firmicutes and Bacteroidetes phyla	Bacterial isolates	*E. coli* NCIMB 12212, *B. subtilis* IA40, *S. aureus* NCIMB 9518, *K. marxianus* CB86556	[93]
*P. boletiformis*, *A. dissimilis* and *H. simulans*	*Pseudovibrio* spp.	Bacterial isolates	*E. coli*, *S. Typhimurium*, *B. subtilis*, *S. aureus*, *S. aureus* MRSA, *S. aureus* VISA, hVISA, *C. perfringens*, *C. difficile*, *Y. enterocolitica*, *B. cereus*, *E. faecium*, *Enterococcus* (VRE) and *L. monocytogenes*	[94]
*Ircinia* sp.	*Vibrio* sp., *Aeromonas* sp., *Bacillus* sp., *Corynebacterium* sp., *Pseudomonas* sp., *Streptococcus* sp., *Enterococcus* sp., *Neisseria* sp., *Veillonella* sp., *Citrobacter* sp. and *Klebsiella* sp.	Bacterial isolates	*A. hydrophila*, *B. subtilis*, *E. durans*, *S. lentus*, *K. pneumoniae* and *R. solanacearum*	[95]
*O. rotunda*		Methanol extract	*B. cereus*, *E. Cloacae*, *E. Coli*, *L. Monocytogenes*, *M. Flavus*, *P. Aeruginosa*, *S. Typhimurium* and *S. aureus*	[96]

**Table 3:** Source, extract/compound, pathogens tested and corresponding references are reported.

Source	Extract/Compound	Pathogens Tested	Reference
*I. variabilis*, *I. spinosula* and *I. oros*	Aqueous, ethanol and dichloromethane extract	*A. coffeaformis*, *P. tricornutum*, *C. Closterium*, *E. intestinalis*, *U. lactuca* and *S. muticum*	[101]
*F. reticulata*	Tryptamine and 6-bromo-8,1’-dihydro-isoplysin A and 5,6-dibromo-8,1’-dihydro-isoplysin A	*V.carchariae* and *V. natrigens*	[99]
*N. chaliniformis*	Ethyl acetate and aqueous extracts	*B. cereus*, *B. pumilus*, *B. megaterium*, *P. haloplanktis*, *P. chlororaphis*, *P. putida*, *P. aeruginosa* and *B. amphitrite*	[104]
*L. isodictyalis*	Ethyl acetate extract	*B. amphitrite*	[105]
*A. cavernosa*	Kalihinenes X, Y and Z	*B. amphitrite*	[106]
*Callyspongia* spp. and *C. plicifera*	Dichloromethane extract	*B. amphitrite* and *H. elegans*	[107]
*G. barretti*	Barretin and 8,9-dihydrobarretin	*B. improvisus*	[100]
*I. basta*, *P. purpurea* and *A. rhax*	Bastadins 3, 4, 9, bastadin-16, hemibastadin-1, aplysamine-2, psammaplin A	*B. improvisus*	[98]
*T. rubra*, *T. maza*, *H. heliophile and P. citrina*	Acetone/dichloromethane extract	*P. perna*	[108]
*Haliclona* sp.	Two hexapeptides	*M. edulis galloprovincialis*	[109]

**Table 4:** Source, extract/compound, activity and corresponding reference are reported.

Source	Sponge Host	Extract/Compound	Activity	Reference
*P. simplex*		Plakortin and Plakortide Q	Antiplasmodial	[111]
*Hymeniacidon* sp.		Monoamphilectine A	Antiplasmodial	[78]
*M. unguiculata*		Ptilomycalin F and Fromiamycalin	Antiplasmodial	[112]
*F.reticulata*		8-oxo-tryptamine and (*E*)-6-bromo-20-demethyl-30-*N*-methylaplysinopsin and (*Z*)-6-bromo-20-demethyl-30-*N*-methylaplysinopsin	Antiplasmodial	[99]
*H. intestinalis*	Bacterial colonies THB20 and THB34	Ethyl acetate extract	Antiplasmodial	[113]
*P. johnstonii*		Pachymatismin	Antileishmanial	[114]
*H. exigua*		Araguspongin C	Antileishmanial	[115]
*Hyrtios* sp.		Hyrtiodoline A	Antitrypanosomial	[116]
*A. oroides*		Ethanol extract	Antifungal	[79]
*H. communis*		Untenospongin B	Antifungal	[119]
*S. carnosus* and *Leucosolenia* sp.	Psedoalteromonas, Bacillus, Vibrio and Staphylococcus phyla	Isolated of bacteria	Antifungal	[93]
*S. officinalis*	*Bacillus* 2011SOCCUF3	Methanol extract	Antifungal	[88]
*N. exigua*		Methanolic extract	Antifungal	[77]
*S. fortis*		Peptide C	Antifungal	[81]
*P. angulospigulatus*		Plakortide N and Plakortide F	Antifungal	[75]
*Plakortis* sp.		Menzamenone M and Menzamenone N	Antifungal	[74]
*E. deficiens*	Proteobacteria, Actinobacteria and Firmicutes phyla	Aqueous extract	Antifungal	[71]
*Psammaplysilla* sp. 1		Ethyl acetate extract	Antifungal	[120]
*C. reniformis*		Collagen extract	Wound-healing	[121]
*I. oros* and *S. foetidus*		Collagen filaments	Wound-healing	[122]
